# Preparation of a nano emodin transfersome and study on its anti-obesity mechanism in adipose tissue of diet-induced obese rats

**DOI:** 10.1186/1479-5876-12-72

**Published:** 2014-03-19

**Authors:** Kun Lu, Shuanshuan Xie, Shilong Han, Jidong Zhang, Xinwen Chang, Jin Chao, Qingqing Huang, Qing Yuan, Haiyan Lin, Lei Xu, Changxing Shen, Min Tan, Shen Qu, Changhui Wang, Xiaolian Song

**Affiliations:** 1 Shanghai Tenth People’s Hospital, Tongji University School of Medicine, Shanghai 200072, China; 2 Department of Molecular and Biomedical Pharmacology, University of Kentucky, Lexington KY 40506, USA; 3 Department of Biological Sciences, Graduate School of Science and Technology, Kumamoto University, Kurokami 8608555, Japan; 4 Department of Chemical Sciences and Nano-biomedicine, Tongji University, Shanghai 200072, China

**Keywords:** Obesity, Nano, Emodin, Transfersome, ATGL, G0S2

## Abstract

**Objective:**

To describe the preparation of nano emodin transfersome (NET) and investigate its effect on mRNA expression of adipose triglyceride lipase (ATGL) and G0/G1 switch gene 2 (G0S2) in adipose tissue of diet-induced obese rats.

**Methods:**

NET was prepared by film-ultrasonic dispersion method. The effects of emodin components at different ratios on encapsulation efficiency were investigated.The NET envelopment rate was determined by ultraviolet spectrophotometry. The particle size and Zeta potential of NET were evaluated by Zetasizer analyzer. Sixty male SD rats were assigned to groups randomly. After 8-week treatment, body weight, wet weight of visceral fat and the percentage of body fat (PBF) were measured. Fasting blood glucose and serum lipid levels were determined. The adipose tissue section was HE stained, and the cellular diameter and quantity of adipocytes were evaluated by light microscopy. The mRNA expression of ATGL and G0S2 from the peri-renal fat tissue was assayed by RT-PCR.

**Results:**

The appropriate formulation was deoxycholic acid sodium salt *vs.* phospholipids 1:8, cholesterol *vs.* phospholipids 1:3, vitamin E*vs.* phospholipids 1:20, and emodin *vs.* phospholipid 1:6. Zeta potential was −15.11 mV, and the particle size was 292.2 nm. The mean encapsulation efficiency was (69.35 ± 0.25)%. Compared with the obese model group, body weight, wet weight of visceral fat, PBF and mRNA expression of G0S2 from peri-renal fat tissue were decreased significantly after NET treatment (all P < 0.05), while high-density lipoprotein cholesterol (HDL-C), the diameter of adipocytes and mRNA expression of ATGL from peri-renal fat tissue were increased significantly (all P < 0.05).

**Conclusion:**

The preparation method is simple and reasonable. NET with negative electricity was small and uniform in particle size, with high encapsulation efficiency and stability. NET could reduce body weight and adipocyte size, and this effect was associated with the up-regulation of ATGL, down-regulation of G0S2 expression in the adipose tissue, and improved insulin sensitivity.

## Introduction

Obesity is now recognized as one of the most important public health problems facing the world today. Epidemiological surveys from many countries show that the mean weight of the population is increasing and that the prevalences of clinically-significant overweight and obesity are rising rapidly in adults and, of particular concern, in children and adolescents [1]. Up to one-third of the adults in some westernized countries are obese and over two-thirds in certain smaller populations such as Pacific Islanders; very few countries remain unaffected by obesity [2]. WHO has estimated that 200 million people worldwide were obese in 1995, rising to 300 million in 2000. Revised estimates suggest that 400 million people aged 15 years were obese in 2005, with almost 800 million overweight. By 2015, WHO predicts that these numbers will increase to 2.3 billion overweight and 700 million obese [3].

The molecular biological mechanism underlying the pathogenesis of obesity is fat production more than fat decomposition. When it comes to lipolysis, well-known HSL is not the only lipase involved, as HSL-knockout mice show normal basal lipolysis and some residual response to catecholamines [4]. An important player is a novel lipase, ATGL, which confers most of the residual lipolytic activity in HSL-knockout mice [5]. In mouse adipocytes, HSL and ATGL together account for 95% of total triacylhydrolase activity [6]. At present, the respective roles of HSL and ATGL in human fat cell lipolysis have not been well established. ATGL is activated by a cofactor, comparative gene identification (CGI)-58. Because CGI-58 interacts with perilipins on the surface of the lipid droplets, a complex inter-play has been proposed between ATGL, perilipins and CGI-58 [7]. Both PKA and PKG phosphorylate and activate human HSL. Thus, the catecholamine and natriuretic peptide pathways converge on HSL to induce lipolysis inhuman fat cells, while ATGL participates in basallipolysis. In mice, ATGL seems to participate in stimulated lipolysis because ATGL deficiency causes a drastic reduction in stimulated lipolysis and ATGL is required for PKA-stimulated lipolysis [4].

G0S2 is a negative regulator of ATGL that mediates fat decomposition through biding to ATGL. G0S2 is highly expressed in adipose tissue and cell differentiation. The results of analysis on GOS2 function in cell cultures in vitro showed that G0S2 directly binds to ATGL, thus inhibiting the activity of TAG hydrolase and attenuating ATGL-mediated fat decomposition [8]. Over expression of G0S2 in lipid droplets of HELA cells could prevent ATGL-mediated fat degradation. In addition, G0S2 inhibits TAG hydrolysis of ATGL through interacting with ATGL via its hydrophobic region and patatin-like region of ATG. Endogenous G0S2 interference accelerates basic and stimulatory fat decomposition. Over expression of G0S2 decelerates adipose tissue and cell decomposition, and therefore G0S2 plays a role in attenuating the action of ATGL, through which TAG hydrolysis is regulated, or in other words, G0S2 inhibits the TAG hydrolase activity of ATGL [9]. G0S2 also inhibits ATGL-mediated transfer of lipid droplets. These findings provide convincing evidence that G0S2 is an important ATGL-mediated negative regulator of fat decomposition. What we are interested in is whether G0S2 expression is regulated by the newly developed external drugs and further plays a coordinative role in reducing fat by affecting ATGL. The purpose of our study was to confirm the above hypothesis and ascertain whether it is possible to increase fat decomposition by regulating G0S2 and ATGL [10].

There are two conventional modern medicines for the treatment of obesity. One is Sibutramine, which reduces fat by decreasing food intake and promoting heat production; however, it may induce hypertension, tachycardia and anorexia. The other is Orlistat, which is a lipase inhibitor to decrease the absorption of diary fat in the gastrointestinal tract [2]. But as it may induce steatorrhea and fat soluble vitamin deficiency, it has been withdrawn from commercial availability. No other new weight-reduction drug has been approved by the Food and Drug Administration of China, except for a few locally certified trandermal patches and beauty creams. Chinese herbal medicines have good therapeutic effects on obesity. Emodin has been shown to have a weight-reduction effect in obese tats. It can also lower lipid levels and reduce the severity of fatty liver [11]. Studies on pharmacochemistry of emodin showed that it is lipophilic [12]. TCM external therapy is also a means of reducing weight. External medicinal friction and massage also have the weight-reduction effect [13]. Inspired by these findings, we postulated whether it was possible to use emodin as an external rubbing and transdermal drug to regulate ATGL and G0S2 and promote fat decomposition so as to achieve systemic weight reduction, knowing that emodin is lipophilic and can pass through the skin to the subcutaneous fat layer. Compared with oral medications, transdermal drugs are much safer.

**Figure 1:**
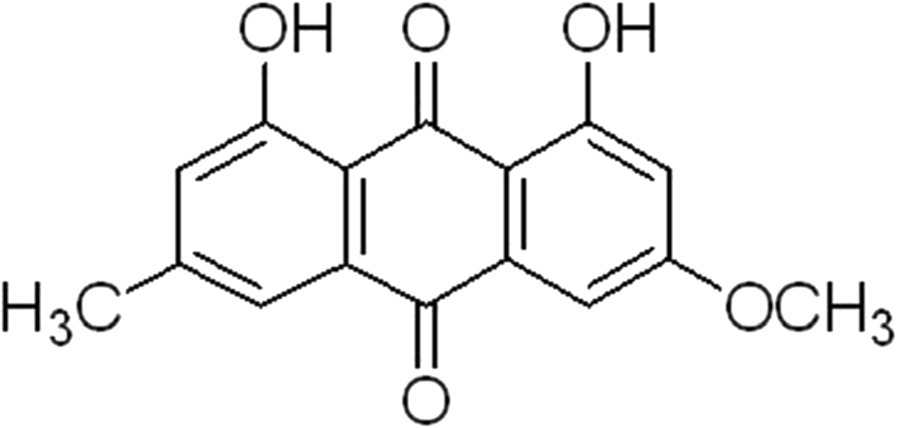
The molecular formula picture of emodin.

**Figure 2:**
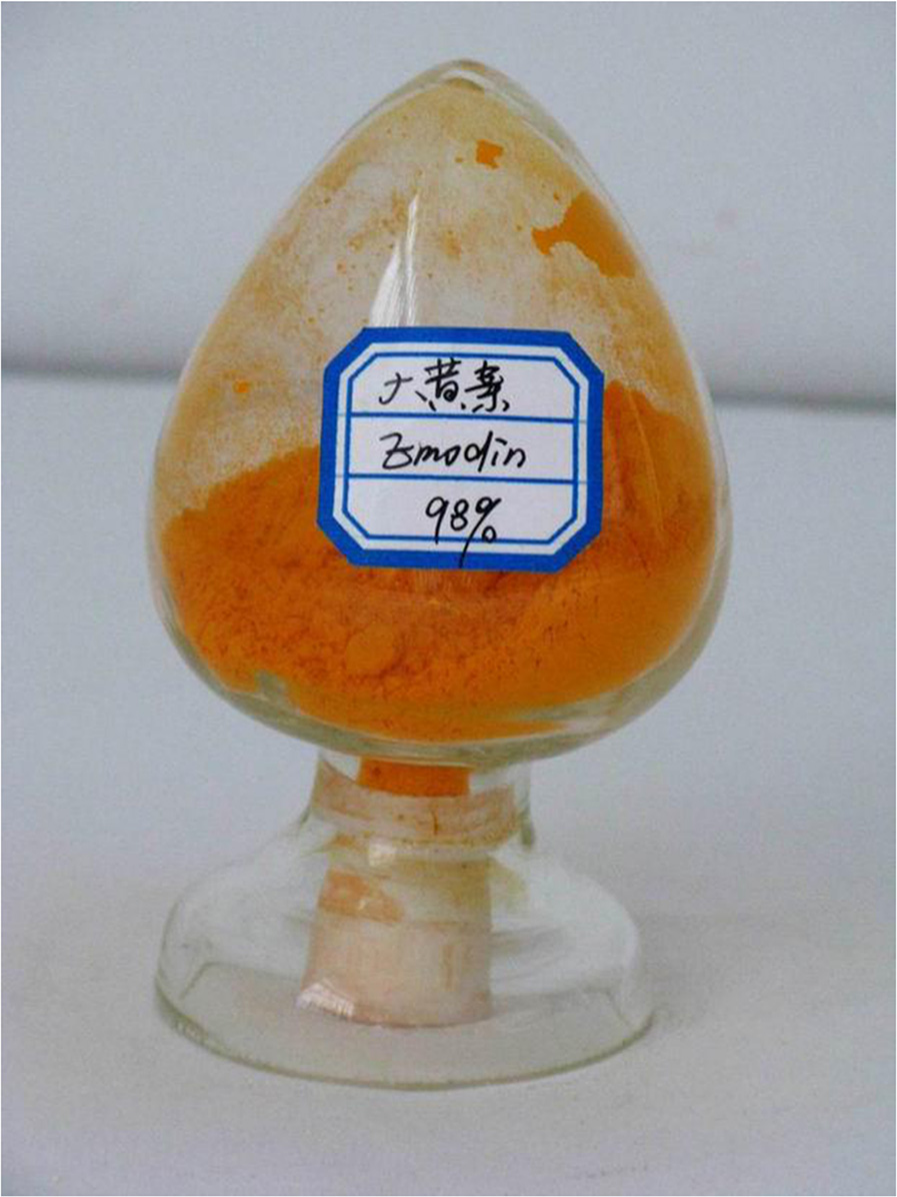
The picture of the traditional chinese herb emodin ingredient.

Emodin is one of the ingredients of Chinese traditional herbs, and its molecular formula is C_15_H_10_O_5_, and is reported to have notable effects on inflammation, hepatocyte injury and liver fibrosis etc (see Figures 1 and 2). In China, emodin has been used as a clinical medication to treat other diseases such as viral hepatitis, cardiac arrhythmia and skin inflammations. In addition, the mitigation of emodin has a protective effect on gastric mucosa and certain protective effects against constipation [11]. Recent evidence indicates that emodin can significantly inhibit the weight gain of high-fatted rats, regulate blood lipids and blood glucose metabolism disorders, enhance anti-lipid peroxidation and protect the liver function [12]. As mentioned above paragraph, it is postulated that emodin may also play an important role in the treatment of obesity by lipolysis and inhibiting proliferation of adipocytes.

Transfersomes are new dose forms developed rapidly in recent years. They can effectively penetrate the skin that is several times larger than themselves, with high permeability and flexibility. The transfersome structure is similar to the biological membrane and can serve as a multi-functional directional drug vector. Sufficiently deformable transfersome can penetrate or cross the intact skin barrier [14]. They were first developed as a carrier preparation in the 1960s, and clinically used in the 1990s [15]. In this study, transfersomes as medicine carriers could expand the therapeutic index of the medicine and reduce the possible adverse effect by improving skin permeation and keeping drug in the skin, which is the advantage of this study compared with others reported liposomes [16]. Otherwise, the poor skin permeation of emodin was a challenge to the development of emodin transdermal delivery system, which is the problem of current preparation technology for emodin nano-liposomes. The resulting drug delivery can be varied systematically: depending on the precise application conditions and carrier design, between 100% and ≤5% of applied drug is deposited in the outermost skin region [17,18]. Owing to the good skin penetration capability of the transfersomes, we are in the position to low the applied doses and nevertheless achieve intra-cutaneous drug concentration and distribution profile qualitatively [19]. We have accumulated some experience in the research and preparation of transfersomes and nano-liposomes [20].

Therefore, in the present study, we attempted to use emodin to prepare a nano transfersome emulsion and make a pharmacological study on it in accordance with the requirements on new nano transfersome emulsions. At the same time, we intended to use the nano emodin transfersome (NET) emulsion for external rubbing massage of the abdomen in a rat obesity model induced by high-fat chows to observe its weight-reduction effect and its effect on the mRNA and protein expressions of ATGL and G0S2 that regulate fat decomposition.

## The pharmaceutical experiments

### Materials and instruments

#### Materials

Lecithin was procured from Lipoid Corporation. (Germany, Batch number: 790618-1/902), Deoxycholic acid sodium salt and Cholesterol were from Guangfu Fine Chemical Co., Ltd. (Tianjin, China). emodin was obtained from Xi’an Haijia Biotechnology Co., Ltd (Batch number: 519-02-8). emodin (reference substance) was purchased from National institute for the control of pharmaceutical and biological products. Sephadex G-50 was purchased from Beijing Haidian Huiyou fine chemical plant. Phosphate butter (PBS) was prepared in our laboratory. All other chemicals were of analytical reagent grade and were used without any further purification.

### Instruments

NET was prepared in Rotary evaporator on the model N1001 and Digital water bath on the mode SB1000 of Shanghai Eyela Corporation. Transmission electron microscope (TEM) was performed on the model H7500 of Hitachi Co., Ltd. Zetasizer analyzer on the model Delsa Nano was purchased from Beckman coulter. lnc (USA). Organic syringe filters (13 mm × 0.22 um) were purchased from Tianjin Dongkang Technology Co., Ltd. Ultrasonic cleaner on the model SB100DT was obtained from Ningbo scientz biotechnology Co., Ltd. YL9100 HPLC chromatograph was provided by Hefei Guochang Analytical Instruments Co., Ltd. Hypersil ODS-2 column (250 mm × 4.6 mm, 5 um) was prepared by Shanghai Troody technology Co., Ltd. The variable-wavelength UV detector (Model 756PC) was purchased from Shanghai Spectrum Co., Ltd.

### Methods and results

#### Preparation method of nano emodin transfersomes (NET)

To prepare the NET with reverse-phase evaporation method, we dissolved sufficient quantum of lecithin, cholesterol in ether, and emodin in 5 ml PBS (aqueous solvent: organic solvent = 1:3 ~ 1:6). The solution was transferred into a pear shape flask, and introduction 10 min after ultrasonic cleaning to form W/O emulsion cream. The flask was then attached to a rotary evaporator to evaporate the organic solvent under a vacuum at 28°C. Dry the solution until colloidal state film is formed on the inner wall of the flask. Take off the flask, add 6.25 mg deoxycholic acid sodium salt and l0 ml PBS (pH 7.2) into the flask. Connect the flask to the rotary evaporator again, rotate under the 35°C and atmospheric pressure with the speed of 40 r/rain, and make it into suspension. The transfersomes suspension was obtained after sonicated for 15 min in ultrasonic cleaner and filter through microporous membrane about 0.22 um.

#### The standard curve of emodin

**Figure 3:**
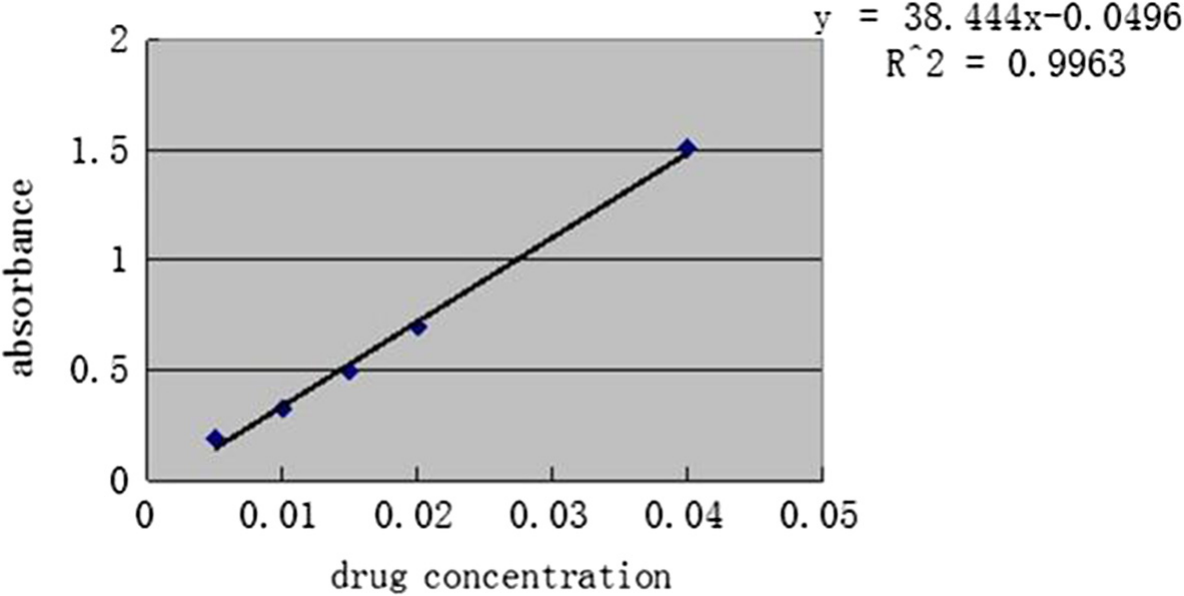
The standard curve of emodin by UV method.

Dissolve 10 mg emodin reference substance in 10 mL distilled water as the standard solution (1 mg/mL), then precisely scale 0.6, 0.8, 1.0, 1.2, 1.4 mL to volumetric flasks and added into mobile phase to 10 mL. Respectively inject 20 uL into ODS-2 column from 0.06, 0.08, 0.10, 0.12, 0.14 mg/mL obtained solution. Take absorbance A as X-axis and drug concentration C (mg/mL) as Y-axis in Figure 3 to determine the regression equation that is:A = 38.444C - 0.0496 (r =0.9963). The result indicates that emodin has a good linear relationship between the scale of 0.005 ~ 0.04 mg/mL.

#### Determination of the entrapment efficiency of NET

The encapsulation efficiency was measured by RP-HPLC. HPLC conditions were as follows: a Hypersil ODS column (250 mm × 4.6 mm, 5 um), room column temperature, the mobile phase consisting of 0.1% H_3_PO_4_ solution: acetonitrile (95:5) (the pH 3.13 adjusted with triethylamine) [21]. The injection volume was 20 uL, the flow rate was 1.0 mL/min, and the UV wavelength was set at 215 nm. The free emodin was separated by Sephadex G-75 column from NET. An aliquot of 0.5 mL transfersomes suspension was added into molecular sieve chromatography on a Sephadex G-75 column (300 mm × 26 mm) which was previously equilibrated with PBS (pH 7.0) [22]. PBS (pH 7.0) was used as eluent and the flow rate was 1.0 mL/min. The emodin peak and transfersomes peak were collected and RP-HPLC was used to determine peak areas. The entrapment efficiency was calculated according to the following equation:

$$E\%=1－\left(M_{\mathrm{free}}/{\mathrm{NET}}_{\mathrm{otal}}\right)\times 100\%$$

Where NET_otal_ was the total amount of drug in transfersomes suspension, and M_free_ was the amount of drug which is not encapsulated in transfersomes [23]. Choose the best formulation and measure the average entrapment efficiency of NET, the result was (69.35 ± 0.25)%.

#### Select the optimum formula of NET

Orthogonal design was used to optimize the preparation methods on the basis of single factor selection. List 4 investigative factors which played important role in the encapsulation efficiency of NET: the weight ratio of cholesterol and phospholipids (A), the emodin dosage (B), the pH value of PBS (C) and the ether volume (D), and respectively design 3 levels in each factor [24] (see Table 1).

**Table 1:** Factors and levels of the orthogonal test

**Levels**	**A**	**B**	**C**	**D**
	**Cholesterol/phospholipids (mg/mg)**	**Emodin dosage (mg)**	**pH value of PBS**	**Ether volume (ml)**
1	1:1	10	6.8	15
2	1:2	20	7.0	20
3	1:4	30	7.2	25

#### Results of L_9_ (3^4^) orthogonal experiment

The L_9_ (3^4^) orthogonal Table 2 was designed to arrange experiment procedures. The content of emodin was used as quality control standards and the encapsulation efficiency as evaluation index. Table 2 illustrated that the degree of impact on the entrapment efficiency were as follows: factor C > A > D > B. Statistical analysis showed that A has a significant difference, C, D have difference, B has little effect. So we choose C3A2D2B2, and the appropriate formulation was as follows: the weight ratio of cholesterol and phospholipids was 1:2, the amount of emodin was 10 mg, the pH value of PBS was 7.2 and the ether volume was 25 mL.

**Table 2:** **Results of L**_**9 **_**(3**^**4**^**) orthogonal experiment of prescription**

**Levels**	**A**	**B**	**C**	**D**	**Encapsulation efficiency (%)**
	**Cholesterol/phospholipids (mg/mg)**	**Emodin dosage (mg)**	**PH value of PBS**	**Ether volume (ml)**	
1	1	1	1	1	45.56
2	1	2	2	2	53.12
3	1	3	3	3	61.34
4	2	1	2	3	58.91
5	2	2	3	1	70.78
6	2	3	1	2	63.71
7	3	1	3	2	64.48
8	3	2	1	3	52.01
9	3	3	2	1	48.91
K1	53.34	56.32	53.76	55.01	
K2	64.47	58.64	53.65	60.44	
K3	55.13	57.99	65.53	57.42	
R	11.13	2.32	11.89	5.35	

#### Shapes and sizes of the NET

**Figure 4:**
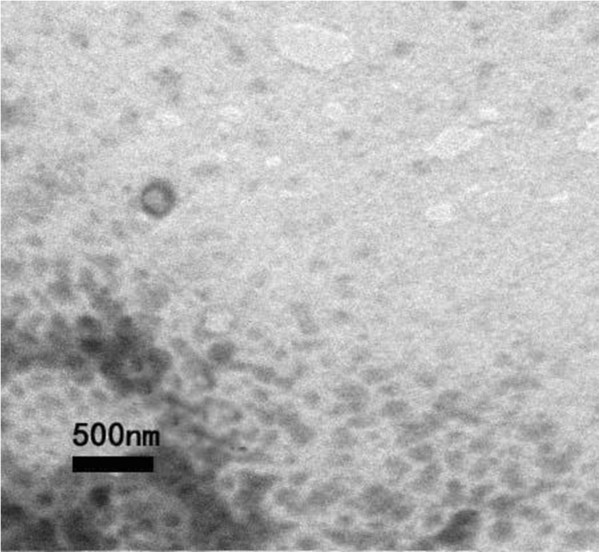
TEM photograph of nano emodin transfersomes.

Dilute stock sample with PBS, then use negative staining sample by 2% phosphotungstic acid (PTA), and observe the shape of transfersomes under TEM (see Figure 4). Suspension of NET was round and uniform and appeared milky.

**Figure 5:**
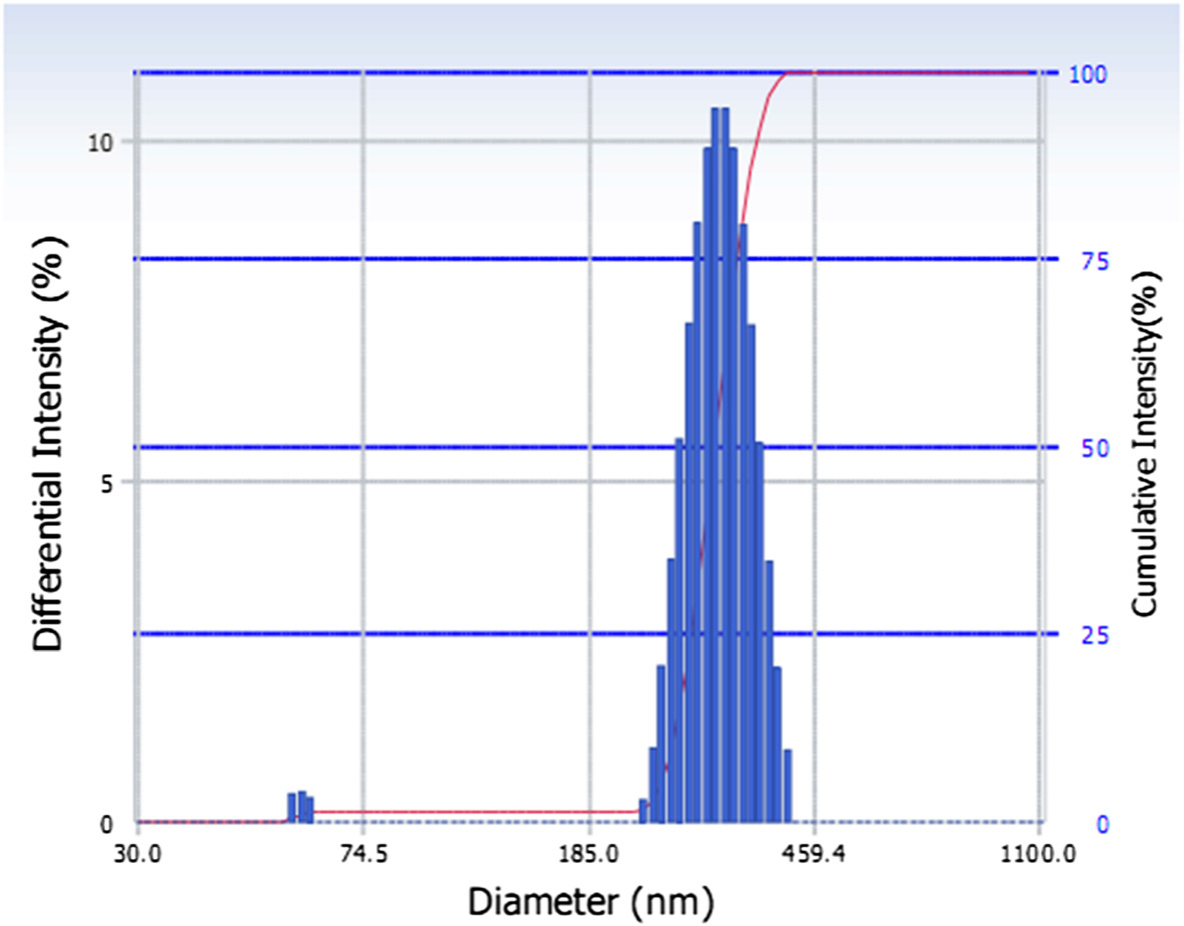
Size distribution of nano emodin transfersomes.

**Figure 6:**
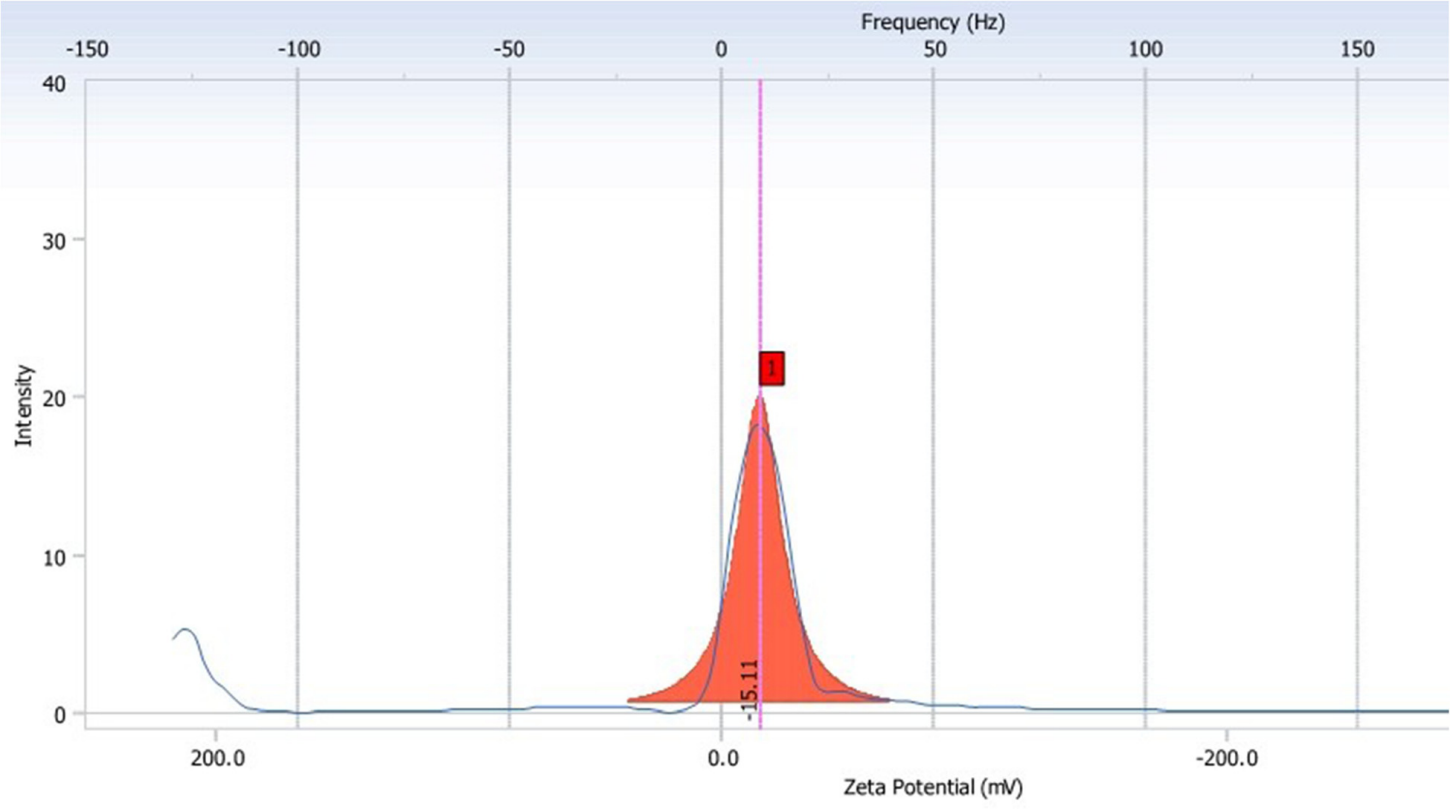
Zeta potential and mobility distribution of NET.

**Figure 7:**
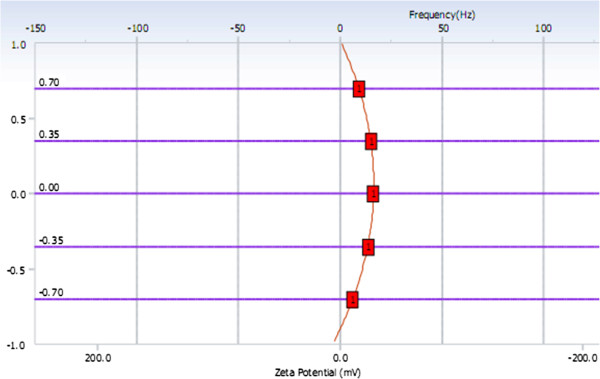
Measurement results of EOS Plot.

The average size of NET determined by Zetasizer and particle analyzer was 292.2 nm, and the polydispersity index was 0.009. The result of size distribution was shown in Figure 5. The Zeta potential was −15.11 mV, as shown in Figures 6 and 7.

### Stability experiment of the NET

Store NET samples at room temperature (25°C) and refrigerator (4°C) in three months, respectively, then we observed the shape and dispersity of transfersomes, measured the entrapment efficiency of NET samples. In cold storage conditions, we found that transfersomes samples, which placed in one month, were not changed in appearance, placed in two months occured statified phenomenon, but gently shaked samples could back to the colloid state as usual. And at room temperature storage in a month, transfersomes appeared irreversible precipitation phenomenon, the particle size turned bigger. These results illustrated that, under room temperature conditions, transfersomes were instability, appeared precipitation, and in cold storage conditions, the particle size had increased, but still accord with the requirement. Indicating that transfersomes need to be placed in cold storage conditions [25]. The different results of entrapment efficiency and particle size were shown in Table 3.

**Table 3:** Entrapment efficiency (EE) and particle size of transfersomes under different conditions (mean ± SD)

**Time (month)**	***±*** **25°C**		***±*** **4°C**	
	**Size (nm)**	**EE (%)**	**Size (nm)**	**EE (%)**
0	297.4	68.83	297.4	68.83
1	463.3	53.46	348.6	67.80
2	675.4	41.09	376.8	66.04
3	713.9	34.78	383.7	65.31
4	-	21.17	390.1	62.68

## The animal experiment

### Materials and methods

#### Experimental materials

##### Reagents and drugs

Oligo(dT) primers, dNTP and MMLV reverse transcriptase were purchased from Dongyang Bio-Technology Co. (Shanghai, China), Trizol from Waston Bio-Engineering Co. (Shanghai, China), and RNase inhibitor from Lingfei Company (Wuhan, China). Taq polymerase and DNA marker were obtained from Tiangen Biochemical Technology Co. (Beijing, China). Primers were synthesized by Yingjun Biotechnology Co. (Shanghai, China).

##### Animals and chow

All animal studies were carried out in compliance with the Principles for Care and Use of Laboratory Animals approved by the Laboratory of Tongji Medical College, China. 60 clean grade just weaning SD male rats, with weight of 50 ± 5 g, were purchased from the Experimental Animal Center of Wuhan University, China. The formula of standard rat chow contained the standard powder 35 g, wheat bran 15.5 g, soybean meal 20 g, corn flour 20 g, soybean oil 0.5 g, fish meal 5 g, bone meal 2.5 g, yeast extract 1 g, and salt 0.5 g in each 100 g, with total energy 14.03 KJ/g ( protein 20.90%, fat 10.38%, and carbohydrate 68.72%). The formula of high-fat diet was given as follows: each 100 g contains standard rat diet 60 g, lard 15 g, dried egg yolk powder 10 g, skim milk 8 g, casein 5 g, sugar 2 g, total energy 19.22 KJ/g (protein 19.45%, fat 49.85%, carbohydrate 30.70%) (20). The feed was mixed by adjustable high-speed electric homogenizer (FSH-2, Jiamei Instrument Co., Jiangsu, China) and stored at room temperature. A week worth of the feed was prepared at a time.

#### Experimental methods

##### The topical emulsion of nano emodin transfersome

The nano emodin transfersome (NET) prepared as described in Part One of the present study was added to glycerol and sodium carboxymethyl cellulose at a weight ratio of 30:3:1, ground and mixed well, and finally prepared into a nano emodin transfersome emulsion for topical use.

##### Preparation of emodin oral solution

Emodin oral solution was composed of Chinese herbal medicine emodin (Hubei) in a 1.5:1 (w/w) ratio. All the herbal drugs were purchased from Ginkgo Traditional Chinese Herbal Company (Wuhan, China). All the herbal drugs were soaked in 8 volumes of distilled water (1:8, w/v) for 12 h at room temperature, following by boiling for 2 h three times. The mixture was filtrated and concentrated to a concentration equivalent to 2.7 g raw herbs in 1 mL extract. The extract was stored at 4°C until use.

##### Grouping and induction of obesity

All rats were randomly assigned to one of two groups, the model group (n = 50) or the normal control group (n = 10). After adaptation with free access to regular rat chow and tap water for one week, 40 rats in the model group were fed with high-fat diet, 25 g for each rat per day, and 10 rats in the normal group were fed with standard rat chow. All rats were maintained in the animal facility individually and housed in a controlled room, with a temperature of 20 ± 5°C, humidity of 55 ± 5%, and 12 h-dark and 12 h-light cycle. The criterion for judging successful induction of obesity was 20% or more increase in body weight as compared with the average weight of the control group. 39 rats in the model group that met the criterion were randomly divided into 4 groups: the model group (n = 9), topical NET group (n = 10), the oral emodin solution group (n = 10) and the massage group (n = 10).

##### Treatment and drug delivery methods

At first, the abdominal hairs of all animals were faded away using 8% sodium sulfide, and then intervened by application of the drug. Animals in the blank control group were massaged with the same amount of distilled water on the abdomen around the acupoint “Shenjue” and “Zhongwan” in a clockwise direction and a circling way at a rate of 60 circles per minute, knowing that these two acupoints have the effect of regulating the endocrine function as recorded in traditional Chinese medicine. Each episode lasted 2 minutes × 3 at a 1-minute interval. The rats in the emodin oral solution group received emodin solution at 2.7 g/kg/day, those in the NET group received topical emulsion of NET at 4 g/kg/day, and those in the model group and the normal group, emodin oral solution group received topical distilled water of the same volume. The exterior daub number is the same as the topical NET group. The treatment administration was performed at 8:30 a.m. every day for 8 weeks. The fur, feces, food intake, vitality and activities of rats were observed daily, and the rats were weighed once at 8:00 a.m. every other day by using an electronic analytical balance with 0.1 g accuracy (M248886, Zhongxi Chemical Glass Instrument Co., Beijing, China).

### Measurement program of parameters

#### Measurement of body weight and obesity parameters

At the end of the treatment, rats were weighed and sacrificed, and 2 mL fasting blood (fasting for 12 h) was taken. The serum samples were collected by centrifuging the blood samples at 3 000 rpm for 10 min (Certrifuge-5415R, Eppendorf Co., Germany). The liver and retroperitoneal, epididymal adipose tissues were removed and weighed (wet weight of visceral fat) after excess blood and tissue fluid were dried by filter paper. PBF was calculated (PBF = wet weight of visceral fat/body weight × 100%).

#### Measurement of blood biochemical parameters

Serum triglycerides (TG), total cholesterol (TC), high-density lipoprotein cholesterol (HDL-C) and low-density lipoprotein cholesterol (LDL-C) were measured using an automatic biochemical analyzer (AU-400, Olympus Co., Japan). The serum insulin was determined by the method of radioimmunoassay. The radioactive count of sediments was determined by using a radioimmunoassay calculator (GC-911, Science and Technology Industrial Corporation of CUST, China).

#### Light microscopy of adipose and liver tissue

A small block of adipose tissue was dissected from the left epididymis and merged in 4% paraformaldehyde for 24 h. The fixed adipose tissue was embedded in paraffin and sliced for HE staining. The quantitative analysis on the size of adipocytes was conducted by an automatic medical color image analysis system (HM IAS-2000, Champion Co., China). The light microscopy protocol of liver tissue are the same steps.

#### Statistical analysis

Statistical analysis of data was performed with the one-factor analysis of variance (SPSS so ware, version 13.0, SPSS Inc). The results were expressed as mean SD (standard deviation), and P < 0.05 was considered to be statistically signicant. All statistical tests were two-sided.

## Results

### The body weight changes of rats after high-Fat diet

**Figure 8:**
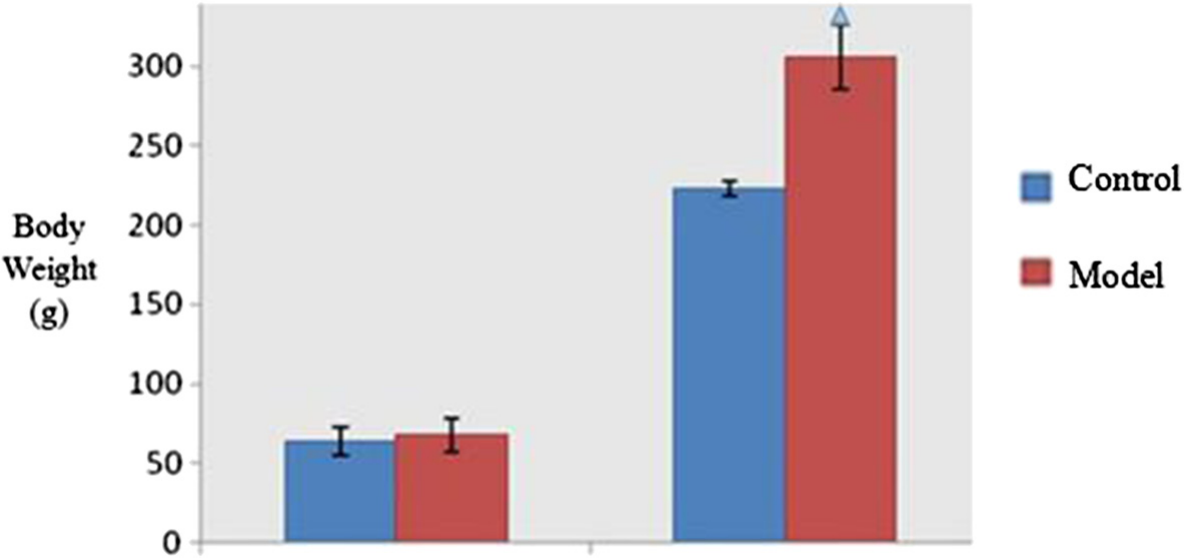
**Body weight changes after high-fat diet.** Note: ^△^*P <* 0.01, as compared with the control group.

The body weight was similar between the normal control group and the obese model group before initiation of the high-fat diet (*P >* 0.05). At the end of 6 weeks on the high-fat diet, the body weight of obese rats in model group was significantly increased compared with that of the normal group (*P <* 0.01, see Figure 8). Besides, there is no change in food intake and physical movement which have been measured upon different treatments.

#### Comparison of general obesity parameters

Compared with the normal group, there was significant difference in body weight between the groups before intervention (P < 0.05. The body weight of the model group was still higher than that of the normal group by the end of treatment (P < 0.05). Compared with the model group, the body weight of the massage, oral and NET groups reduced significantly (P < 0.05). Compared with the massage group, the body weight of the oral and NET group reduced significantly (P < 0.05), and there was no significant difference in weight reduction between the oral and NET groups (P > 0.05). There was no significant difference in liver wet weight between the groups (P > 0.05). The liver wet weight of all intervention groups was higher than that of the normal group (P < 0.05), and the liver wet weight of the oral group was lower than that of the massage and NET groups (P < 0.05). There was no significant difference in the body fat ratio between the groups (P > 0.05) (see Table 4).

**Table 4:** **Comparison of general obesity parameters among groups (**$\bar{x}$**± S)**

**Group**	**N**	**Body weight (g)**		**Liver weight (g)**	**Wet weight of visceral fat (g)**	**PBF (%)**
		**Before treatment**	**After treatment**			
Control	10	222.89 ± 5.28	327.33 ± 18.18	13.46 ± 5.58	6.89 ± 1.86	2.37 ± 0.44
Model	9	295.34 ± 16.61^△^	442.44 ± 31.23^△^	12.84 ± 1.71	9.67 ± 1.76^△^	2.42 ± 0.40
Massage	10	309.04 ± 30.18^△^	423.13 ± 58.99^△^	12.05 ± 1.56	9.94 ± 2.40^△^	2.44 ± 0.49
Oral	10	314.83 ± 15.25^△^	378.28 ± 28.79^△▲☆^	13.45 ± 1.39	8.16 ± 1.87^△▲☆^	2.35 ± 0.37
NET	10	305.06 ± 24.10^△^	383.175 ± 33.65^△▲☆^	12.82 ± 1.59	9.91 ± 2.79^△★^	2.38 ± 0.99

**Notes: **^△^*P <* 0.05, compared with the control group; ^▲^*P <* 0.05, compared with the model group; ^☆^*P <* 0.05, compared with the Massage group; ^★^*P <* 0.05, compared with the Oral group.

#### Comparison of serum lipids parameters

There was no significant difference in serum TC and LDL between the groups (P > 0.05). Compared with the model and massage group, the body weight of the oral and NET group reduced significantly after treatment (P < 0.05), and there was no significant difference between the NET and oral groups (P > 0.05). There was no significant difference in TG between the model and massage groups (P > 0.05). HDL level of the NET and oral groups was higher than that of the normal, model and massage groups (P < 0.05), and there was no significant difference between NET and oral groups (P > 0.05) (Table 5).

**Table 5:** **Comparison of TG, TC, HDL-C and LDL-C among groups (**$\bar{x}$**± S)**

**Group**	**n**	**TG (mmol/L)**	**TC (mmol/L)**	**HDL-C (mmol/L)**	**LDL-C (mmol/L)**
Control	10	1.01 ± 0.19	2.15 ± 0.23	1.58 ± 0.24	0.52 ± 0.21
Model	9	1.31 ± 0.22^△^	2.18 ± 0.43	1.41 ± 0.32	0.49 ± 0.29
Massage	10	1.23 ± 0.12^△^	1.98 ± 0.31	1.43 ± 0.28	0.57 ± 0.28
Oral	10	0.86 ± 0.27^▲☆^	2.04 ± 0.40	2.76 ± 0.38^△▲☆^	0.47 ± 0.20
NET	10	0.99 ± 0.27^▲☆^	2.01 ± 0.20	2.67 ± 0.31^△▲☆^	0.53 ± 0.21

**Notes:**^ △^*P <* 0.05, compared with the control group; ^▲^*P <* 0.05, compared with the model group; ^☆^*P <* 0.05, compared with the Massage group.

#### Comparison of general features of the epididymal adipocytes

**Figure 9:**
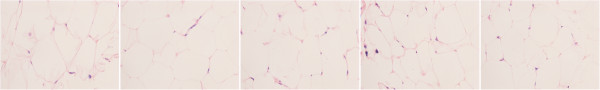
Histological changes of epididymal adipose tissue in rats (HE Stain, × 400).

The equivalent diameter, perimeter and area of adipocytes in the model group significantly increased compared with the normal control group (*P <* 0.01), and the number of adipocytes decreased as quantified in a single field under × 400 original magnification (data not shown, see Figure 9). All values of the Oral group and NET group resulted in significant reduction in comparison to that of untreated obese rats in the obese model group (*P <* 0.01, *P <* 0.05, respectively), with greater a reduction for all parameters for the oral group (see Table 6).

**Table 6:** **Comparison of equivalent diameter, perimeter and area of adipocytes among proups (**$\bar{x}$**± S)**

**Group**	**n**	**Equivalent diameter (um)**	**Perimeter (um)**	**Area (um**^**2**^**)**
Control	10	54.81 ± 9.78	180.42 ± 27.75	2304.43 ± 763.34
Model	9	79.87 ± 14.67^△^	297.32 ± 54.60^△^	5134.45 ± 1689.43^△^
Massage	10	75.61 ± 12.43^△^	283.06 ± 40.60^△^	4912.98 ± 1588.21^△^
Oral	10	52.87 ± 18.45^▲☆^	171.32 ± 35.78^▲☆^	2237.12 ± 687.02^▲☆^
NET	10	59.98 ± 16.65^▲☆^	225.21 ± 65.87^▲☆^	2982.98 ± 1931.98^▲☆^

**Notes: **^△^*P <* 0.01 compared with the control group; ^▲^*P <* 0.05 compared with the model group; ^☆^*P <* 0.05, compared with the Massage group.

#### Histomorphological observation on the liver

**Figure 10:**
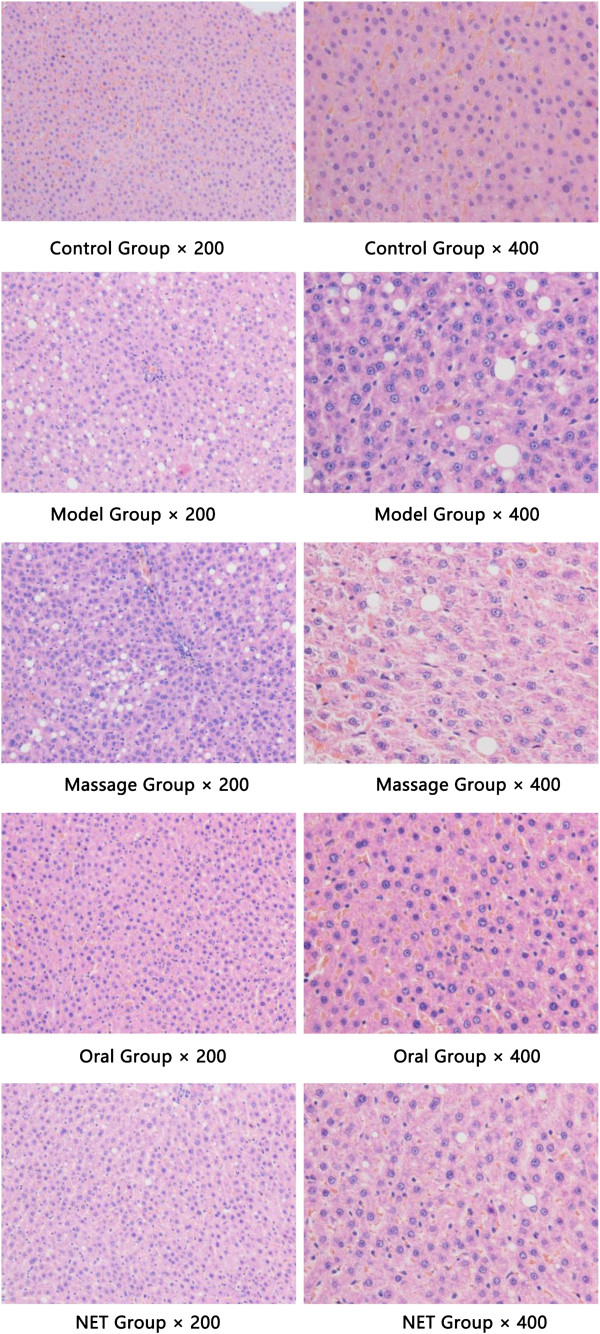
Histomorphological observation on the liver tissues (HE Stain, × 200 and × 400).

The liver tissue was HE stained and observed under an optical microscope (× 200 and × 400). It was found that the structure of the hepatic lobules was intact in the normal group, with radiating arrangement of hepatic cell cords around the central vein, and clear and uniform Disse’s spaces; hepatocytes were relatively large in size and polygonic in shape, with rich cytoplasm and large nuclei in the center without steatosis seen. In the model and massage groups, the structure of hepatocytes and Disse’s spaces were not clear enough; large numbers and different sizes of circular vacuoles were seen in the cytoplasm, presenting as macrovesicular steatosis. In the oral group, the hepatocytes were structurally intact and arranged in a radiating way, with clear and uniform Disse’s spaces and nuclei; a small amount of macrovesicular steatosis was vaguely seen in the cytoplasm. In NET group, hepatocytes were structurally intact, with clear and uniform Disse’s spaces and nuclei, presenting as several steatotic vacuoles (see Figure 10).

#### Comparison of ATGL and G0S2 protein expression in adipose tissue

**Figure 11:**
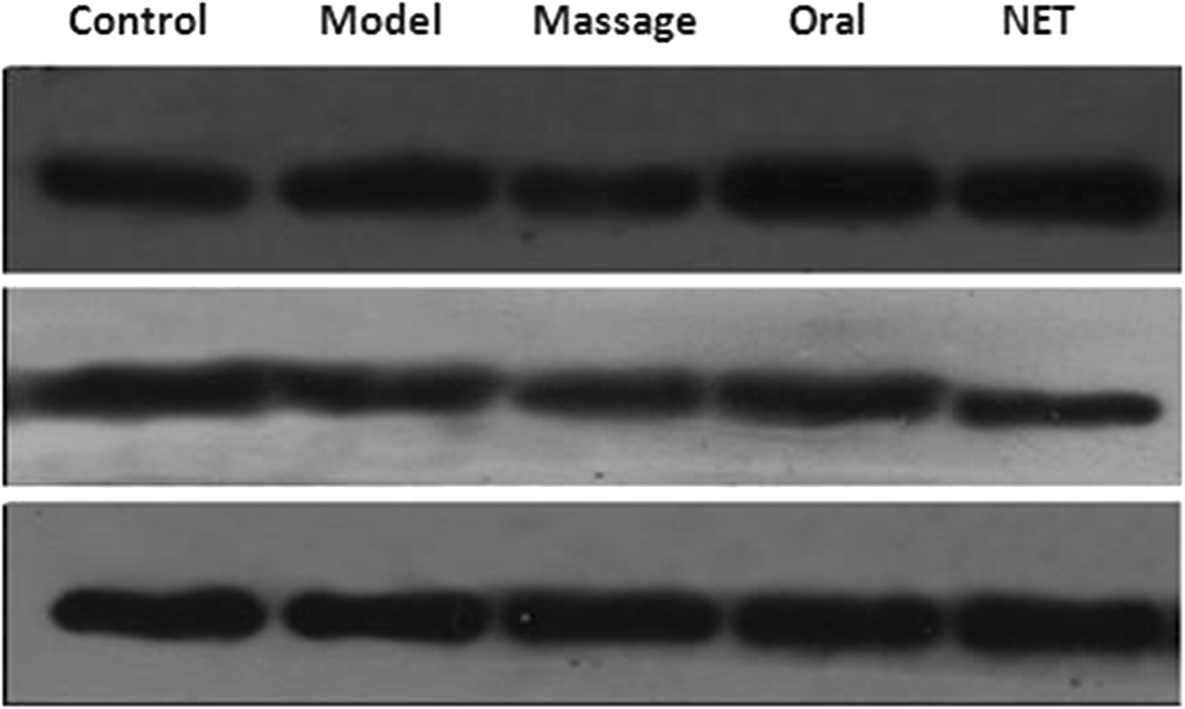
ATGL and G0S2 protein expression in the adipose tissue by Western-blot.

**Figure 12:**
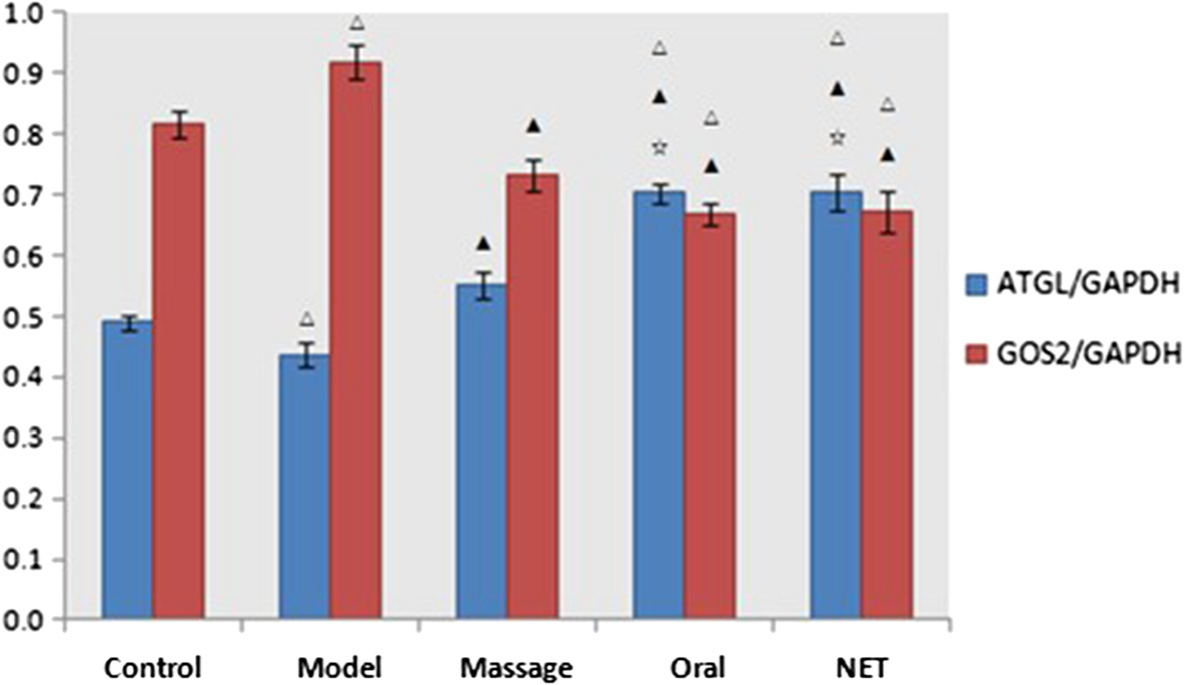
**Comparison of ATGL and G0S2 protein expression in the adipose tissue.** Notes: ^△^*P <* 0.05 compared with the control group; ▲*P <* 0.05 compared with the model group; ^☆^*P <* 0.05, compared with the Massage group.

Compared with the normal group, the expression level of ATGL protein was slightly lower and the expression level of G0S2 protein was higher in the adipose tissue of the model group (both P < 0.05). The expression level of ATGL protein in the massage, oral and NET groups was high than that in the model group (P < 0.05). The expression level of ATGL protein in the oral and NET group was higher than that in the massage group (P < 0.05). The expression level of G0S2 protein in the massage and NET groups was lower than that in the model group (P < 0.05), and the expression level of G0S2 protein in the oral and NET groups was lower than that in the massage group (P < 0.05) (see Table 7 and Figures 11, 12).

**Table 7:** Comparison of ATGL and G0S2 protein expression in the adipose tissue

**Group**	**n**	**ATGL**	**G0S2**
Control	10	0.590 ± 0.011	0.716 ± 0.022
Model	9	0.437 ± 0.020^△^	0.918 ± 0.028^△^
Massage	10	0.552 ± 0.023^▲^	0.732 ± 0.025^▲^
Oral	10	0.702 ± 0.016^△▲☆^	0.667 ± 0.018^△▲^
NET	10	0.705 ± 0.031^△▲☆^	0.671 ± 0.035^△▲^

**Notes: **^△^*P <* 0.05 compared with the control group; ^▲^*P <* 0.05 compared with the model group; ^☆^*P <* 0.05, compared with the Massage group.

## Conclusions

The treatment of obesity is confronted with a stern challenge at present. With the commercial withdrawal of Sibutramine and Orlistat, no effective and low-toxicity new weight-reduction drug has been developed and marketed. Our and other studies have demonstrated that Matrine could reduce the body weight and Lee’s indexes of obese rats, as well as fasting insulin, TG and cholesterol levels [17]. By taking advantage of the TCM theories, our study not only holistically confirmed that Matrine could reduce the body weight and abdominal fat weight of obese rats, decrease the size of fat cells and increase the HDL-C level but gained insights into the mechanism of Matrine in affecting the adipose tissue of obese rats. Based on our previous work, the present study, for the first time, used the thin-film dispersion method to successfully prepare a nano NET emulsion. The obtained TF carry negative charges and are small in size and well distributed, with good entrapment efficiency and stability. The result of external application showed that it was able to relieve the pathological change of fatty liver, reduce the peripheral fat content, increase serum HDL-C, and reduce TG level. In addition, it could also down-regulate the G0S2 protein expression in the adipose tissue of obese rats and up-regulate the ATGL protein expression. These mutually antagonistic effects work together to reduce the body weight of obese rats. There is no similar report available in the literature. The mechanism of NET in the prevention and treatment of obesity needs to be further studied, knowing that regulation of G0S2 and ATGL may prove to be a new strategy for the treatment of obesity.

The development of obesity is attributed to multiple factors responsible for disordered homeostasis of fat metabolism, increased fat synthesis and decreased fat decomposition in the body. Studies have demonstrated that multiple factors participate in and regulate fat metabolism. ATGL and G0S2 are believed to play an important role in regulating fat metabolism. ATGL is a key enzyme in fat metabolism, and is expressed mainly in murine white and brown fat tissues, and secondarily in the cardiac muscle, skeletal muscle and testis [5,9]. ATGL mainly hydrolyzes triglyceride into diglyceride, and therefore ATGL and HSL are believed to be a common rate-limiting enzyme for the reaction of fat decomposition. The adipose tissue was increased remarkably in ATGL knockout mice, presenting as TG accumulation in multiple tissues, indicating that ATGL is a rate-limiting enzyme of TG hydrolysis, playing an important role in fat mobilization in fat tissues [26-28]. Other studies also reported that G0S2 is a negative regulator of ATGL [28]. Zandbergen et al. reported that the level of G0S2 mRNA expression was the highest in fat tissues, and was up-regulated in the process of preadipocyte differentiation into fat cells [29]. In vitro and cell-based studies on G0S2 function showed that G0S2 directly binds to ATGL, thus decreasing the ability of ATGL-mediated fat decomposition by inhibiting the activity of TAG hydrolase [30].

In the present study, we established a rat model of alimentary obesity induced by feeding the animals with well-prepared high-fat chows for two weeks. After 6-week feeding, the body weight of the animals in the model group was 20% heavier than that in the normal group. In addition, both wet weight ratio and body lipid ratio of the internal organs of the animals in the model group were increased. HE staining also showed that fat cells became significantly larger as shown by optical microscopy. All these indicate the successful establishment of the obesity model. Animal studies have demonstrated that the expression of ATGL mRNA was down-regulated markedly in db/db and ob/ob obesity animal models, suggesting that the development of obesity may be associated with the decreased level of ATGL [6,25]. The result of the present study showed that the expression level of ATGL protein in the model group was slightly lower than that in the normal group, which may be the reason for the increased fat synthesis and excessive accumulation of fat in the body, resulting in obesity. Our finding is grossly consistent with the results of other studies.

The present study holistically confirmed that NET could reduce body weight and abdominal fat weight of obese rats. The biochemical parameters also showed that TG was decreased and HDL-C was increased after topical treatment with NET. Compared with the model group, the equivalent diameter, circumference and area of fat cells in the intervention group were also decreased significantly. Pathological observation showed that the liver tissue was normal, suggesting that NET emulsion may affect the process of energy consumption in fat metabolism. In summary, ATGL and G0S2 play an important role in energy metabolism of the body. In the present study, we used the WB technique to further observe its impact on ATGL and G0S2 protein expression in the adipose tissue of obese rats. The result showed that NET was able to down-regulate G0S2 protein expression in the adipose tissue of obese rats, and up-regulate ATGL protein expression to exert its weight-reduction effect. The above pharmodynamic study demonstrated that GOS2 and ATGL could regulate fat metabolism, which is consistent the previous studies [31-33]. We also testified the effect of G0S2 in attenuating the action of ATGL in rats in vivo. The blood biochemical parameters, pathological observation of the fat and liver tissue, and molecular biological parameters obtained in our study indicate that NET emulsion had a good weight-reduction therapeutic effect equivalent to that of oral emodin, which is also consistent with our previous study [34]. But the therapeutic effect of the external NET emulsion is better than that of massage in reducing body weight.

Emodin is a kind of water soluble molecules and hard to be entrapped. Transdermal drug delivery system has been accepted as potential non-invasive route of drug administration, with advantages of avoidance of the first-pass metabolism, sustained therapeutic action and better patient compliance, though, its prevalent use is restricted due to excellent impervious nature of skin. It is the greatest challenge for researchers to surmount the inherent limitations imposed by stratum corneum of skin, for enhanced transdermal drug delivery to achieve systemic therapeutic concentration [35]. Transfersomes have shown immense potential in drug delivery across the skin [36]. Since the transfersomes has high hydrophilicity and flexibility. In the role of hydration transfersomes could across l/10 ~ l/4 of itself diameter of the channel, made loaded drugs through the skin barrier and even enter into the blood circulation. Thus, preparing NET is a good way to improve its entrapment efficiency.

The preparation method by reverse-phase evaporation was simple and had high encapsulation efficiency, easy for scale up preparation and research [37]. We choosed the deoxycholic acid salt as surfactant, when the weight ratio of deoxycholic acid salt and phospholipids was 1:8, the NET had well stability and high entrapment efficiency. The experiment indicated that encapsulation efficiency was the highest when the weight ratio of cholesterol and phospholipids was 1:2, the amount of emodin was 10 mg, the pH value of PBS was 7.2 and the ether volume was 25 mL. RP-HPLC is the most widely used separation technique for this application owing to its simplicity and general applicability to emodin type alkaloids [38], which could determine entrapment efficiency accurately as well as emodin quantity. The average entrapment efficiency of NET was 61.46 ± 0.25% by RP-HPLC determination. Zeta potential is one of the important indexes of the surface charge of ion, and also affected the stability of transfersomes suspension liquid [39,40] transfersomes with negative potential can reduce mutual gather and fusion, increase the stability. The Zeta potential of NET prepared in this experiment was −7.94 mV. And the particle size of NET with negative charge was small and uniform, and had some stability. The above methods for preparation of NET and determination of its physicochemical properties, provide excellent separation and good precision, and are relatively straight forward and reliable for both chromatographic conditions and sample preparation. Furthermore, the study of NET was not comprehensive, and need further investigation.

The results of the present study may also provide some inspirations and references for further research on the preparation of nano materials and treatment of obesity, and for the development of new weight-reduction drugs as well.

## Competing interests

This study was supported by the National Natural Science Foundation of China (No. 81172229, 81100018). The funders had no role in study design, data collection and analysis, decision to publish, or preparation of the manuscript. All authors declare that they have no competing interests of the final published manuscript.

## Authors’ contribution

KL, SX, SH, CJ, QH, JZ, CW, SQ, Nz, CS, HL, LX, QY, MT and XS had role in study design, data collection and analysis, decision to publish and preparation of the manuscript. KL, SX, SH, CW and XS carried out the studies, participated in the design of the experiment. KL, JZ, CS, HL, LX, QY and MT draft and revise the manuscript. Both authors read and approved the final manuscript.
